# Enhanced reptile search optimization with convolutional autoencoder for soil nutrient classification model

**DOI:** 10.7717/peerj.15147

**Published:** 2023-04-07

**Authors:** Prabavathi Raman, Balika J. Chelliah

**Affiliations:** Department of Computer Science and Engineering, SRM Institute of Science and Technology, Ramapuram Campus, Chennai, Tamil Nadu, India

**Keywords:** Convolutional autoencoder, Hyperparameter tuning, Machine learning, Reptile search optimization, Soil nutrients

## Abstract

**Background:**

Soil nutrients play an important role in soil fertility and other environmental factors. Soil testing is an effective tool for evaluating soil nutrient levels and calculating the appropriate quantitative of soil nutrients based on fertility and crop requirements. Because traditional soil nutrient testing models are impractical for real-time applications, efficient soil nutrient and potential hydrogen (pH) prediction models are required to improve overall crop productivity. Soil testing is an effective method to evaluate the presence of nutrient status of soil and assists in determining appropriate nutrient quantity.

**Methods:**

Various machine learning (ML) models proposed, predict the soil nutrients, soil type, and soil moisture. To assess the significant soil nutrient content, this study develops an enhanced reptile search optimization with convolutional autoencoder (ERSOCAE-SNC) model for classifying and predicting the fertility indices. The model majorly focuses on the soil test reports. For classification, CAE model is applied which accurately determines the nutrient levels such as phosphorus (P), available potassium (K), organic carbon (OC), boron (B) and soil pH level. Since the trial-and-error method for hyperparameter tuning of CAE model is a tedious and erroneous process, the ERSO algorithm has been utilized which in turn enhances the classification performance. Besides, the ERSO algorithm is derived by incorporating the chaotic concepts into the RSO algorithm.

**Results:**

Finally, the influence of the ERSOCAE-SNC model is examined using a series of simulations. The ERSOCAE-SNC model reported best results over other approaches and produces an accuracy of 98.99% for soil nutrients and 99.12% for soil pH. The model developed for the ML decision systems will help the Tamil Nadu government to manage the problems in soil nutrient deficiency and improve the soil health and environmental quality. Also reduces the input expenditures of fertilizers and saves time of soil experts.

## Introduction

One of the primary sources of income and occupations for a large group of people in India is agriculture. There was an exponential rise in the demand for production over the years. However, with the surge in industrialization, there comes a vast decline in the agricultural lands (Wankhede, 2020). To make precise decisions depends on the crop type which has to be planted and gains a good yield, data like usage of fertilizers, pesticides, meteorological, and soil information has to be made available to the agriculturalists promptly and accurately. Better crop production is reached by farmers *via* study of the appropriate circumstances, thus, minimizing the damages and crop loss that arises because of the unfavorable situations (Motia & Reddy, 2020). Numerous hybrids (Słowik & Cpałka, 2021) and high yielding varieties of plants were produced daily. However, they are input sensitive and need sufficient exogenous inputs to yield the desired output (John et al., 2020). Accurate and timely detection of crop-related problems enables decision makers (agricultural experts) and farmers to make appropriate crop environment management and soil resource management decisions. Soil nutrients are an important property that contributes to soil fertility as well as other environmental factors. According to previous research it could have a significant impact on community biomass, vegetation distribution, species composition, and plant size. As a result, an efficient method for evaluating soil nutrients is required for increased agricultural productivity. To meet the crop needs the application of synthetic and chemical inputs caused harm by making more ecological degradation (Suchithra & Pai, 2020). Prevention of losses becomes the key factor for many artificial methods. However, crop loss is reduced, and yield is increased by agronomists who have a thorough understanding of several factors. Soil nutrition is critical to soil fertility and the ecological conditions for plant growth and development (Trontelj Ml & Chambers, 2021). Moreover, several research works have endeavored to exploit and better quantify the significance indulged in soil nutritional conditions variation.

Even though noticeable developments were done in understanding the relationships among plant growth and soil nutrition, research scholars were uncertain regarding the response of every soil nutrient which is available since it can be based on its quality and content grade, and it has strongly species-specific and varies among congeners (Bondre & Mahagaonkar, 2019). Thus, the correct prediction of soil nutrient quality becomes highly important for conducting research on the growth of endangered tree species and forest regeneration. One more research point is determining a suitable soil category for machine learning (ML) without substantial accuracy loss (Wu et al., 2020). In Ghadge et al. (2018) discussed in this article is predicated on the idea that using machine learning to forecast soil parameters will increase its accuracy. The correlations found in this study are crucial for comprehending the whole method for predicting soil parameters with optical spectroscopy sensors. Numerous study findings have been presented and examined. Additionally, when choosing between 3-level, 5-level, or 13-level nutrient characterization for particular nutrients, which can be employed for a more exact nutritional characterization technique, the influence of category levels was not as substantial as expected. A comparison between soil from a nearby farm with a similar texture and soils taken from several areas in Slovenia was done, and the results provided a better prediction for a nearby farm.

In Garg et al. (2019) soil analysis is an appropriate technique to assess soil quality. In laboratories, soil is examined for analysis, which results in reports with disorganized and pointless data. This study uses a variety of big data analysis and machine learning techniques to extract knowledge from the data and identify fertilizer recommendation classes based on the current soil nutrition composition. The Tata soil and water testing centre provided the soil analysis reports needed for this experiment. The performance of artificial neural networks (ANN) and stochastic gradient descent (SGD) on the Hadoop platform are analyzed in this article using the Mahoot library. DL applications generally decline the dependence on spatial-form models and pre-processing methods by simplifying end-to-end procedure that happens directly from input images (Vahl de Paula et al., 2020). Among several DL models, convolutional neural network (CNN) proves to be one such accepted network model.

Convolutional autoencoders (CAEs) (Xu & Duraisamy, 2020) use convolutional layers to filter out noise and create robust and stable feature representations while reducing the input dimension size, making them suitable for dealing with high-dimensional noisy images. It is worth noting that one advantage of CAEs over traditional dense autoencoders for image processing is that when stacking and slicing the data, a significant loss of information is observed. Instead of stacking the data as in traditional autoencoders, the convolutional layers of CAEs can efficiently retain the spatial information of the input image data and extract information gently. In other words, CAEs, like other CNNs, can learn compressed image latent representations preserving the spatial locality of the input (Wickramasinghe, Marino & Manic, 2021).

This study develops an enhanced reptile search optimization with convolutional autoencoder for soil nutrient classification (ERSOCAE-SNC) model. The presented ERSOCAE-SNC model aimed to categorize the nutrient levels of P, K, OC, and B. For soil nutrient classification, CAE model is applied which accurately determines the nutrient levels of the soil. Finally, the ERSO algorithm with the combination of chaotic concept and RSO algorithm is used as hyperparameter optimizer of the CAE model. Finally, a hyperparameter optimizer based on the ERSOCAE-SNC technique was developed. A comprehensive simulation analysis was performed on benchmark datasets to investigate the improved prediction results of the ERSOCAE-SNC technique.

## Materials and Methods

### Machine learning for soil nutrient classification

Keerthan Kumar, Shubha & Sushma (2019) Proposed based on error analysis, the system employs supervised machine learning algorithms such as linear regression (LR) multi-variate, support vector machine (SVM), and random forest classifier to produce the best results. The results of these algorithms will be compared, and the best one, random forest classifier, which produces the best and most accurate results, will be chosen. As a result, this system will aid in reducing the farmers’ difficulties. Analysis of important soil properties, and based on that, we are dealing with soil grading and crop prediction for the land. In Saranya & Mythili (2020), introduced a technique for categorizing the soil based on the macronutrient and micronutrient and predicting the crop type that is cultivated in specific soil types. Different ML methods are utilized namely bagged tree, k-nearest neighbor (KNN), KNN, LR, and SVM. Kaur & Malik (2021) The purpose of this article is to present a method for developing simple linear regression and multiple linear regression-based models to analyse basic soil macronutrients and micronutrients. The models were used to examine the interdependence of the most important nutrients, namely nitrogen (N), phosphorus (P), and potassium (K), also known as primary nutrients of soils, as well as to assess the influence of N content on other vital soil nutrients. The results of these three models validated the interdependence of these vital nutrients on one another.

Suchithra & Pai (2020) proposed village-wise soil fertility indices of available phosphorus (P), available potassium (K), organic carbon (OC), and boron (B) and the parameter soil reaction are classified using soil test report values (pH). Classifying and predicting village-wise soil parameters reduces fertilizer waste, increases profitability, saves chemical soil analysis experts’ time, and enhances soil health and environmental quality. Extreme learning machine (ELM) with gaussian radial basis, sine-squared, hyperbolic tangent, triangular basis, and hard limit activation functions solves these five classification tasks. After the performance analysis of ELMs with different activation functions for these soil parameter classifications, the Gaussian radial basis function performs best for four out of five problems and exceeds 80% in most accuracy rate calculations in every problem, followed by hyperbolic tangent, hard limit, triangular basis, and sine-squared. Chambers (2021) proposed a study based on hypotheses that ML approaches improve the precision of predictions of soil properties. The relationship established in this work is important for comprehending the overall strategies for soil property prediction using an optical spectroscopy sensor. Investigates various ML models such as RF, decision tree (DT), naive Bayes (NB), SVM, least squares SVM (LSSVM), and artificial neural network (ANN).

Rajamanickam (2021) presents various supervised ML models such as DT, KNN, and SVM for predicting soil fertility based on micro and macronutrient status established in datasets. A supervised ML algorithm is used on training datasets and tested on test datasets, and R Tools is used to execute this algorithm. Rajamanickam & Mani (2021) proposed predicting soil fertility by integrating uncertainty quantification using fisher ratio pre-processing models and Kullback divergent chi-square FS. Then, rather than an individual value, Gustafson-Kessel probabilistic NN classifications use the soil fertility prediction models to generate the likelihood distribution as output and the distinct types of soil fertility levels. Sirsat et al. (2018) used the most accessible regression method to predict fertility indexes for soil organic carbons and four significant soil nutrients (zinc, manganese, phosphorus pentoxide, and iron), specifically a group of seventy-six regressors from twenty families, involving boosting NN, DL, SVM, RF, bagging, Bayesian models, ridge regression, and so on (Abualigah et al., 2022). The optimal result is obtained by employing extra trees that achieve satisfactory predictive results.

In Kaur & Malik (2021), introduced a model that could explore whether the soil is fertile or not, sowing crop seeds on fertile soil, and finally predict the crop yield on distinct soil characteristics. Based on prediction, it has been recommended and suggested that crops grow faster. Different ML approaches including SVM, RF, NB, LR, MLP, and ANN are utilized for crop yield and soil classification. In Suchithra & Pai (2020), implemented an ML technique for predicting mustard crop yields (Pandith et al., 2020) before from soil analysis. For the experiment analysis, data has been gathered that comprises soil samples of various regions of Jammu for mustard crop. Five supervised ML methods such as KNN, NB, multinomial LR, ANN, and RF were employed on the gathered information.

The Pawar and Chillarge (Chambers, 2021) system has been proposed that could assist the farmers by making them aware of the soil condition. Farmers are maximizing crop yields by knowing nutrient proportion of soils. Soil toxicity (Pawar & Chillarge, 2018) affects the soil nutrient that indirectly affects crop conditions. The suggested technique forecast the toxicity level existing from the soil and make farmer aware of them. The number of farmers is dependent on rainfall which is major factor for poor development and decreases crop yields. Therefore, the presented method suggests to farmers about the water supply, toxicity level, crop, and fertility of soil. In this work, sensor accuracy and classification algorithm are of great importance. In Rajamanickam (2021), efforts have been made to explore the connection between element content in (i) plant leaves grown on the soil, (ii) different soils, and (iii) variations in chlorophyll a fluorescence parameter, to explore a technique for earlier recognition of plant stress resultant in the grouping of nutrient status in natural condition (Kalaji et al., 2018). To accomplish this objective, a mathematical process was utilized that integrates PCA (a tool for data complexity reduction), hierarchical k-means (classification model), and an ML methodology—a super-organizing map.

### Enhanced reptile search optimization with convolutional autoencoder

**Figure 1:**
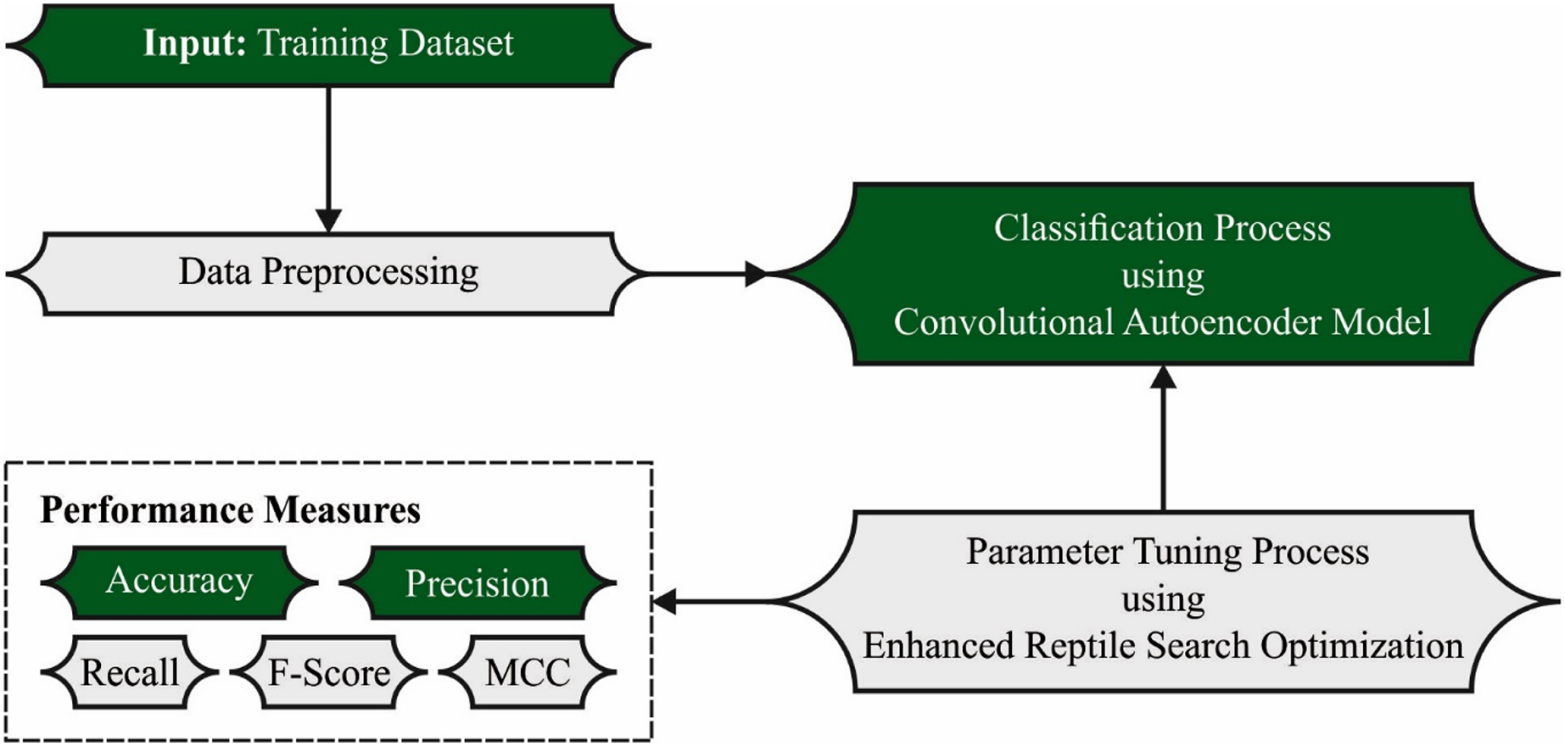
Overall process of ERSOCAE-SNC approach.

In this study, a novel ERSOCAE-SNC model was established for soil nutrient classification. The presented ERSOCAE-SNC model aimed to categorize the nutrient levels of P, K, OC, and B. To accomplish this, the ERSOCAE-SNC model applied a three-stage process namely sample collection, CAE classification, and ERSO based hyperparameter optimization. Figure 1 depicts the overall process of ERSOCAE-SNC approach.

#### Data collection

Soil instances are composed by individual agronomists in soil testing lab. The soil instances were inspected to distinct variables of immediate relevance for plant nutrition namely OC, soil reaction (pH), micronutrients, electrical conductivity (EC), and plant obtainable primary nutrients (P, K). The analytical methods employed for estimating soil fertility. EC is a metric of focus on soluble salt and the degree of salinities from the soil was defined as utilizing of conductivity meter with 1:2.5 soil water suspension. The OC was calculated by Walkley and Black’s wet digestion approach. The phosphorous was evaluated utilizing ascorbic acid model and potassium in soil was defined as the solution ratio of 1:5 of neutral normal ammonium acetate solution and potassium in the extracting was calculated utilizing flame photometry. Afterward, the available boron (B) from soils is extracted with the hot water extraction process. The agricultural data gathered in farmland contains four major parameters such as OC, P, K, and B. All the classes include three subclasses such as medium, high and low.

#### Soil nutrient classification using CAE model

**Figure 2:**
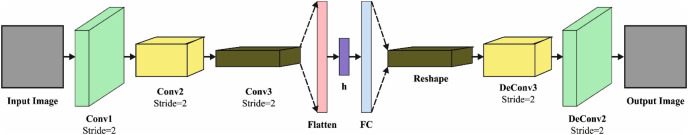
Framework of CAE.

For the classification of soil nutrients such as P, K, OC, and B, the CAE model is exploited in this study. In this study, introduce a new end-to-end convolutional autoencoder (CAE). Also, an attention mechanism is used to improve the accuracy of abundance estimation by making it easier to pull out the diagnostic spectral features of a given endmember. Also, a semi-supervised classification pipeline is introduced that is based on CAE and uses endmember abundance maps as classification features. Real hyperspectral datasets are used for experiments, and supervised and semi-supervised models are used to compare the results (Rajamanickam & Mani, 2021). The layer-wise training is implemented for optimizing parameters of the entire network. In $1D{\rm -}CNN$, a massive amount of data labels is needed in the training phase for parameter optimization leads to the consumption of considerable amount of time. In $1D{\rm -}CAE$, higher level representations of input dataset are extracted in convolution AE without any labels. In finetuning phase, a small amount of data labels is required for classifier construction. There exist two stages in training process of $1D{\rm -}CAE$, that is., fine-tuning (supervised learning) and convolution AE training (unsupervised learning). During the encoder training, AE is stacked in a convolution manner for building the $1D{\rm -}CAE$ method, and the network parameter is enhanced by layer-wise training in unsupervised fashion. Figure 2, showcases the infrastructure of CAE.

The CAE comprises encoding and decoding layers with activation function of Tan-hyperbolic (Tanh). Firstly, the input is mapped into a low dimension space that is later used for reconstructing the primary input in the decoder. In every layer, higher level representation is generated by convolving features of the low layer with kernel learned through a denoising AE. During the AE training, the strong feature representation is produced based on patches extracted from feature maps in the low layer. Using this layer of mapping, the feature representation is extracted from signal process.

The architecture of CAE is instinctively same as AE except for the shared weight in $1D{\rm -}CAE$. CAE applies a $1D$ convolutional kernels for obtaining local feature maps from input vector that gets a non-linear conversion using the subsequent formula:

(1) $$x_j^l = f\left( {\mathop \sum \nolimits_{i \in {M_j}} x_i^{l - 1}\;{\rm *}\;k_{ij}^l + b_j^l} \right),$$

In Eq. (1), ${M_j}$ denotes the input feature maps, $l$ represents the $l$- $th$ layers in $1D{\rm -}CAE$, $ke$ indicate the convolutional kernel of $s \times 1$ and $s$ denotes the size of convolutional kernel. $f$ signifies the activation function, usually a hyperbolic tangent or sigmoid function, and $b_j^l$ is bias vector. Every output feature is produced by certain convolutional kernels.

The output feature maps of convolutional layer are additionally extracted in sub-sampling layers and later the dimension of output features is decreased. The $N$ input mapping generates $N$ output feature. The downsampling is evaluated by the following equation:

(2) $$x_j^l = f\left( {\beta _j^ldown\left( {x_j^{l - 1}} \right) + b_j^l} \right),{\rm \; \; \; \; \; \; \; \; \; }$$

In Eq. (2), down $(.)$ signifies sub-sampling function, $\beta$ and $b$ are product and addition deviations, correspondingly. Input feature map of $n \times 1$ is included by down $(.)$ that decreases the size of output feature to $1/n.$ Down sampling and convolution operations finish encoder on the input vector. $CAE$ employs decoder and encoder in a convolution manner for extracting higher level feature representation of input dataset. During the decoder stage, the feature representation is used for re-producing the original input through de-convolution function such that the procedure noise is additionally decreased. During the encoder stage, the input feature map is convolved by $1D{\rm \; }$ convolution kernel through the conversion of Tanh activation function that eliminates the redundant data for obtaining the feature representation.

The output layer with $T$ neuron is added to the top of $CAE$ for implementing classifier tasks with cascaded feature maps ${f_v}$ from the preceding layer as input, and the output is evaluated by the following equation:

(3) $$0 = f\left( {{b_0} + {w_0}{f_\nu }} \right){\rm \; \; \; \; \; \; \; \; \; \; \; \; }$$

In Eq. (3), ${b_0}$ and ${w_0}$ indicates bias and weight vectors, correspondingly. The learning parameter $k_{ij}^l,$ $b_j^l$ and ${b_0}$ are augmented by gradient descent in the BP process. In comparison to the feedforward BP network, the shared weight of the convolution layer reduces the amount of network parameters that facilitate to prevent the vanishing gradients. The loss function of $CAE$ is shown below:

(4) $$Loss = avg|X - {X^ - }{|^2}{\rm \; \; }$$

In Eq. (4), $X$ and $\bar X$ represents the input and output of encoder, correspondingly. The gradient descent approach is implemented for minimizing the reconstruction.

#### Hyperparameter tuning

At the final stage, the ERSOCAE-SNC model applied the ERSO approach to fine tune the hyperparameters of the CAE technique. RSO algorithm is simulated by the hunting performance of crocodiles. Two important stages of crocodile performance were executed like encircling that is applied by high walking or belly walking, and hunting that is executed by hunting co-ordination or hunting co-operation. Other metaheuristic optimization algorithm performs well, but it has drawbacks such as reduced population diversity and unbalanced exploitation and exploration capabilities. This article proposes RSO, a modified variant of RSO, to improve the performance and search capability of existing optimization method. An adaptive chaotic reverse learning strategy is proposed to optimize from the initialization and in each iteration update to improve population diversity. An elite alternative pool strategy was developed to balance exploitation and exploration. To modify all the individuals and guide the evolutionary direction, a shifted distribution estimation strategy was used. RSO performance is fully validated using 23 benchmark functions, IEEE CEC2017 benchmark functions, and robot path planning problems. A convergence analysis, stability analysis, and statistical tests show that the proposed algorithm is superior (Sirsat et al., 2018).

Parameter initialization: the control parameter and the algorithmic parameter must be initialized beforehand implementing the RSO. The control parameter includes (N) that characterizes the number of crocodiles, and (T) as the maximal iteration count. Further, two algorithmic variables are applied in RSO namely $\alpha$ and $\beta$. The two algorithmic variables are utilized for controlling exploration and exploitation capabilities, correspondingly, to reach an accurate balance among the two capabilities in the searching technique.

Initialization of $RSA$ population. In this stage, we arbitrarily produce a set of initial solutions as follows:

(5) $${X_{i,j}} = X_j^{{\rm \; min\; }} + rnd \times \left( {X_j^{{\rm \; max\; }} - X_j^{{\rm \; min\; }}} \right),\, \forall i = 1,2,\,\ldots, N,\,\forall j = 1,2,{\rm \; } \ldots ,d,$$

In Eq. (5), ${X_{i,j}}$ characterizes the decision parameter of $i$- $th$ solution at $j$- $th$ location. The upper and lower limits of the decision parameter at $j$- $th$ location are $x_j^{{\rm \; max\; }}$ and $j{X^{{\rm \; min}}}$. rnd indicates an arbitrarily produced number within zero and one, $d$ shows the overall amount of decision parameters at every solution. The $N$ solution set is produced and saved in X:

(6) $$X = \left[ {\matrix{ {{X_{1,1}}} \hfill & {{X_{1,2}}} \hfill & \ldots \hfill & {{X_{1,d{\rm -}1}}} \hfill & {{X_{1,d}}} \hfill \cr {{X_{2,1}}} \hfill & {{X_{2,2}}} \hfill & \ldots \hfill & {{X_{2,d{\rm -}1}}} \hfill & {{X_{2,d}}} \hfill \cr \vdots \hfill & \vdots \hfill & \ldots \hfill & \vdots \hfill & \vdots \hfill \cr {{X_{N,1}}} \hfill & {{X_{N,2}}} \hfill & \ldots \hfill & {{X_{N,d{\rm -}1}}} \hfill & {{X_{N,d}}} \hfill \cr } } \right],$$

In Eq. (6), every row ${X_i} = \left( {{X_{i1}},{\rm \; }{X_{i,2}},{\rm \; } \ldots ,{\rm \; }{X_{i,d - 1}},{\rm \; }{X_{i,d}}} \right)$ specifies the solution of ${i^{th}}$ positions.

Fitness calculation. The fitness values of every solution in the population must be evaluated by $f\left( {{X_i}} \right)$ $\forall i = 1,2,$ $\ldots$ , $N.$

Encircling stage: this phase is an exploration behavior of crocodile in RSO designed to find the best solution in the searching space of problem that follows two approaches, such as belly walking and high walking. The belly walking approach is controlled using $Tl4 < t \le 2Tl4$T, whereas the high walking approach is controlled using $t \le Tl4$:

(7) $${X_{i,j}}\left( {t + 1} \right) = \left\{ {\matrix{ {X_j^{Best}\left( t \right) - {\eta _{i,j}}\left( t \right) \times \beta - {R_{i,j}}\left( t \right) \times rnd,t \le \displaystyle{T \over 4}} \hfill \cr {X_j^{Best}\left( t \right) \times {X_{r1,j}}\left( t \right) \times ES\left( t \right) \times rnd,\displaystyle{T \over 4} < t \le \displaystyle{{2T} \over 4}} \hfill \cr } } \right.$$

In Eq. (7), ${X_{i,j}}$ indicates the decision parameter of $i$- $th$ solution at $j$- $th$ location. $X_j^{Best}\left( t \right)$ refers to the ${j^{th}}$ location in the optimal solution at $t$ iterations. $t + 1$ indicates the novel iteration, but the preceding iteration indicates $t$. The hunting operator of $j$- $th$ location in $i$- $th$ solution is indicated as ${\eta _{i,j}}\left( t \right)$ that is evaluated by Eq. (8). The variable $\beta$ control the exploration ability of the high walking approach. The $\beta$ value is fixed as 0.1. $rand$ is an arbitrarily produced number ranging from [0,1]. ${X_{r1,j}}\left( t \right)$ indicates the decision parameter at $j$- $th$ location, where $r1 \in \left[ {1,{\rm \; }N} \right].$ ${\eta _{ij}}\left( t \right),$ ${P_{i,j}}$, and Avg $\left( {{X_i}} \right)$ are evaluated, correspondingly:

(8) $${\eta _{i,j}} = X_j^{Best} \times {P_{i,j}},{\rm \; \; \; }$$

(9) $${P_{i,j}} = \alpha + \displaystyle{{{X_{i,j}} - Avg\left( {{X_i}} \right)} \over {X_j^{Best} \times \left( {X_j^{{\rm \; max\; }} - X_j^{{\rm \; min\; }}} \right) + e^\prime}}{\rm \; }$$

(10) $$Avg{\rm \; }\left( {{X_i}} \right) = \displaystyle{1 \over d}\mathop \sum \nolimits_{j = 1}^d {X_{i,j}},{\rm \; \; \; \; \; \; \; }$$

Now, ${P_{i,j}}$ indicates the percentage difference among the decision parameter at $j$- $th$ location of the optimum solution ${X^{Best}}$ and the decision parameter at similar location to the existing solution ${X_i}.$ $\alpha$ is fixed as 0.1, that is used for controlling the exploration capability of RSO in the hunting cooperation. $\epsilon$ refers to a random number within [0, 2]. Avg $\left( {{X_i}} \right)$ indicates the average values of decision variable of the existing solution ${X_i}.$ ${R_{i,j}}\left( t \right)$ is a factor utilized for decreasing the searching space of $j$- $th$ location in $i$- $th$ solutions and ES (t) indicates the evolutionary sense probability and assigns an arbitrarily reducing number from [2, −2]:

(11) $${R_{i,j}} = \displaystyle{{X_j^{Best} - {X_{r2,j}}} \over {X_j^{Best} + e^\prime}}{\rm \; \; \; }$$

(12) $$ES{\rm \; }\left( t \right) = 2 \times r3 \times \left( {1 - \displaystyle{1 \over T}} \right){\rm \; },{\rm \; \; \; \; \; \; }$$

Now, $r2$ is an arbitrarily produced value ranging from 1 and $N$ that represents the index of one solution in the population *viz*., arbitrarily selected. $r3$ indicates a random number ranging from [−1, 1].

Hunting stage: this phase is exploitation behavior of crocodiles in the RSO designed to exploit the existing research region to $rnd$ the optimum solution based on: hunting cooperation and coordination. The hunting cooperation is controlled using $t \le T$, whereas hunting coordination is controlled using $t \le 3Tl4$.

(13) $${X_{i,j}}\left( {t + 1} \right) = \left\{ {\matrix{ {X_j^{Best}\left( t \right) \times {P_{i,j}}\left( t \right) \times rnd,\displaystyle{{2T} \over 4} < t \le \displaystyle{{3T} \over 4}} \hfill \cr {X_j^{Best}\left( t \right) - {\eta _{i,j}}\left( t \right) \times e - {R_{i,j}}\left( t \right) \times rnd,\;\displaystyle{{3T} \over 4} < t \le T} \hfill \cr } } \right.$$

Termination condition. Repeat from Step 3 to 5 until it reaches the maximal number of iterations $T.$

The ERSO algorithm is derived using chaotic concepts in the RSO algorithm. Optimization algorithm depending on chaos concept uses stochastic search method (Yadav, Chopra & Vijayalakshmi, 2021). The characteristics of chaotic mapping and reverse learning are first used to introduce an adaptive chaotic stochastic search method to increase the population diversity of RSO. Second, to balance the research and development of RSO, an elite alternative pooling strategy is used. Additionally, the evolutionary direction is changed using a distribution estimation strategy. The population direction is better guided by sampling the information from the dominant population, which increases the convergence efficiency of the algorithm. This algorithm is distinct from intelligent population-based algorithms and evolving competitive algorithms.

Because of non-repetitive nature of chaos concept, it carries out the global search at a fast pace when compared to accidental search that is compared with probability. Also, it is guaranteed that the member population covers the whole searching region. As a result, optimum or closer to optimum response would be amongst the population.

One of the popular chaotic maps is the logistic chaotic map. This is a second order polynomial and it can be determined by the following equation:

$${x_{j + 1}} = a{x_j}\left( {1 - {x_j}} \right)$$

(14) $$for{\rm \; }0 < a \le 4{\rm \; }j = 0,1,2, \ldots {\chi _j} \in \left[ {0,1} \right]$$

In Eq. (14), initial value of function is denoted by ${x_0}$; ${x_n}$ refers to the function afterward ${n^{th}}$ iteration. The initial condition needs to be lies within the [0, 1]. $\lambda$ must be fixed to four for making this equation show chaotic behavior. So that ${\rm \; }{x_0} \ne \left\{ {0,0.25,0.5,0.75} \right\}.$

The second system to enhance the RSO algorithm is to utilize chaotic mapping for updating SD formula.

(15) $$\sigma \left( r \right) = {\bigg(\displaystyle{{T - t} \over T}\bigg)^n}\left( {{\sigma _{initial}} - \sigma_{final}} \right) + {\sigma _{final}} \times z\left( t \right)$$

In Eq. (15), $z\left( t \right)$ is corresponding to chaotic mapping in $t$- $th$ iterations.

## Results

This section determines which chaotic mapping sequence to use in conjunction with the adaptive reverse learning strategy after evaluating various chaotic mapping combination algorithms using benchmark test functions. A total of 23 benchmark test functions that are frequently used in the literature are used in this section. Seven unimodal functions, six multimodal functions, and ten fixed dimensional functions are among the benchmark test functions. Using a Python 3.6.5 tool, the ERSOCAE-SNC technique’s (Han et al., 2020) effectiveness is evaluated for classifying soil nutrients and pH levels This section investigates the soil nutrient classification performance of the ERSOCAE-SNC model on a dataset comprising 5,000 samples under five classes of soil nutrients as depicted in Table 1.

**Table 1:** Dataset details.

Class	Sub-class			Total
	Low	Medium	High	
OC-F	500	500	500	1,500
P-F	500	500	–	1,000
K-F	500	500	500	1,500
B_F	500	500	–	1,000
Total number of soil samples				5,000

**Figure 3:**
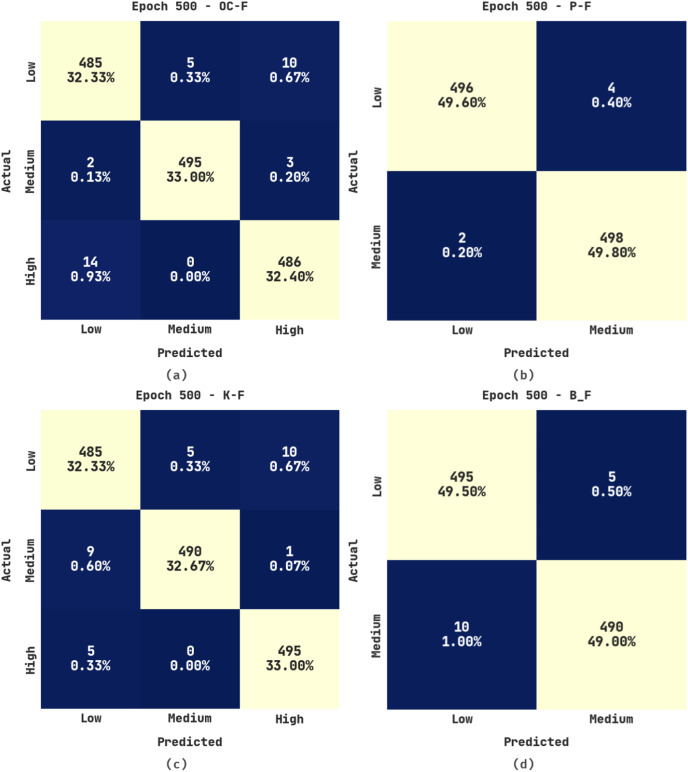
Confusion matrices of ERSOCAE-SNC approach under epoch 500 (A) OC-F, (B) P-F, (C) K-F, and (D) B_F.

Figure 3 shows the confusion matrices produced by the ERSOCAE-SNC model under 500 epochs. With 500 epochs, the ERSOCAE-SNC model has identified the subclasses of OC-F with 32.33% under low class, 33% under medium class, and 32.40% under high class. Eventually, with 500 epochs, the ERSOCAE-SNC approach has identified the subclasses of K-F with 32.33% under low class, 32.67% under medium class, and 33% under high class. Simultaneously, with 500 epochs, the ERSOCAE-SNC technique has identified the subclasses of B_F with 49.50% under Low class and 49% under medium class.

Table 2 offers a brief result analysis of the ERSOCAE-SNC model on the soil nutrient classification process with 500 epochs. The results implied that the ERSOCAE-SNC model has reached effective classification results. For instance, the ERSOCAE-SNC model has recognized samples under OC-F class with $acc{u_y}$, $pre{c_n}$, $rec{a_l}$, ${F_{score}}$, and MCC of 98.49%, 97.73%, 97.73%, 97.73%, and 96.60% respectively. Likewise, the ERSOCAE-SNC system has recognized samples under P-F class with $acc{u_y}$, $pre{c_n}$, $rec{a_l}$, ${F_{score}}$, and MCC of 98.67%, 98%, 98%, 98%, and 97% correspondingly. Meanwhile, the ERSOCAE-SNC technique has recognized samples under B-F class with $acc{u_y}$, $pre{c_n}$, $rec{a_l}$, ${F_{score}}$, and MCC of 98.50%, 98.50%, 98.50%, 98.50%, and 97% correspondingly.

**Table 2:** Result analysis of ERSOCAE-SNC approach with various class labels under epoch 500.

Epoch-500					
Class labels	Accuracy	Precision	Recall	F-score	MCC
Organic carbon-F					
Low	97.93	96.81	97.00	96.90	95.35
Medium	99.33	99.00	99.00	99.00	98.50
High	98.20	97.39	97.20	97.30	95.95
Average	98.49	97.73	97.73	97.73	96.60
Phosphorus-F					
Low	99.40	99.60	99.20	99.40	98.80
Medium	99.40	99.20	99.60	99.40	98.80
Average	99.40	99.40	99.40	99.40	98.80
Potassium-F					
Low	98.07	97.19	97.00	97.10	95.65
Medium	99.00	98.99	98.00	98.49	97.75
High	98.93	97.83	99.00	98.41	97.61
Average	98.67	98.00	98.00	98.00	97.00
Boron-F					
Low	98.50	98.02	99.00	98.51	97.00
Medium	98.50	98.99	98.00	98.49	97.00
Average	98.50	98.50	98.50	98.50	97.00

**Figure 4:**
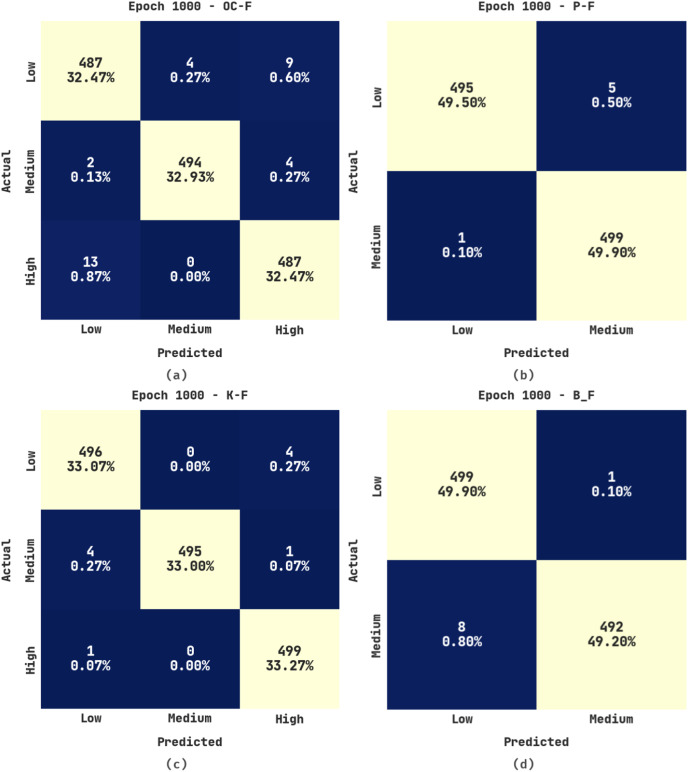
Confusion matrices of ERSOCAE-SNC approach under epoch 1000 (A) OC-F, (B) P-F, (C) K-F, and (D) B_F.

Figure 4 illustrates the confusion matrices produced by the ERSOCAE-SNC approach under 1,000 epochs. With 1,000 epochs, the ERSOCAE-SNC methodology has identified the subclasses of OC-F with 32.47% under low class, 32.93% under medium class, and 32.47% under high class. Also, with 1,000 epochs, the ERSOCAE-SNC system has identified the subclasses of K-F with 33.07% under Low class, 33% under medium class, and 33.27% under high class. At last, with 1000 epochs, the ERSOCAE-SNC algorithm has identified the subclasses of B_F with 49.90% under low class and 49.20% under medium class.

Table 3 provides a brief outcome analysis of the ERSOCAE-SNC technique on the soil nutrient classification process with 1000 epochs. The outcomes exposed that the ERSOCAE-SNC approach has obtained effective classification outcomes. For sample, the ERSOCAE-SNC algorithm has recognized samples under OC-F class with $acc{u_y}$, $pre{c_n}$, $rec{a_l}$, ${F_{score}}$, and MCC of 98.58%, 97.87%, 97.87%, 97.87%, and 96.80% correspondingly. In addition, the ERSOCAE-SNC system has recognized samples under P-F class with $acc{u_y}$, $pre{c_n}$, $rec{a_l}$, ${F_{score}}$, and MCC of 99.56%, 99.34%, 99.33%, 99.33%, and 99% correspondingly. In the meantime, the ERSOCAE-SNC technique has recognized samples under B-F class with $acc{u_y}$, $pre{c_n}$, $rec{a_l}$, ${F_{score}}$, and MCC of 99.10%, 99.11%, 99.10%, 99.10%, and 98.21% correspondingly.

**Table 3:** Result analysis of ERSOCAE-SNC approach with various class labels under epoch 1,000.

Epoch-500					
Class labels	Accuracy	Precision	Recall	F-score	MCC
Organic carbon-F					
Low	98.13	97.01	97.40	97.21	95.80
Medium	99.33	99.20	98.80	99.00	98.50
High	98.27	97.40	97.40	97.40	96.10
Average	98.58	97.87	97.87	97.87	96.80
Phosphorus-F					
Low	99.40	99.80	99.00	99.40	98.80
Medium	99.40	99.01	99.80	99.40	98.80
Average	99.40	99.40	99.40	99.40	98.80
Potassium-F					
Low	99.40	99.00	99.20	99.10	98.65
Medium	99.67	100.00	99.00	99.50	99.25
High	99.60	99.01	99.80	99.40	99.10
Average	99.56	99.34	99.33	99.33	99.00
Boron-F					
Low	99.10	98.42	99.80	99.11	98.21
Medium	99.10	99.80	98.40	99.09	98.21
Average	99.10	99.11	99.10	99.10	98.21

**Figure 5:**
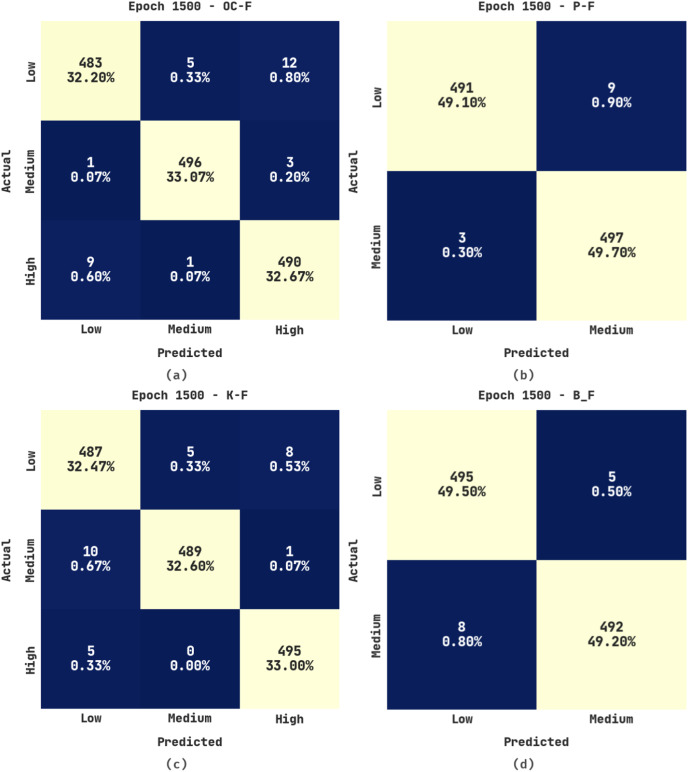
Confusion matrices of ERSOCAE-SNC approach under epoch 1,500 (A) OC-F, (B) P-F, (C) K-F, and (D) B_F.

Figure 5 portrays the confusion matrices produced by the ERSOCAE-SNC approach under 1,500 epochs. With 1,500 epochs, the ERSOCAE-SNC methodology has identified the subclasses of OC-F with 32.20% under low class, 33.07% under medium class, and 32.67% under high class. Besides, with 1,500 epochs, the ERSOCAE-SNC technique has identified the subclasses of K-F with 32.47% under low class, 32.60% under medium class, and 33% under high class. Lastly, with 1,500 epochs, the ERSOCAE-SNC algorithm has identified the subclasses of B_F with 49.50% under Low class and 49.20% under medium class.

Table 4 provides a detailed outcome examination of the ERSOCAE-SNC system on the soil nutrient classification process with 1,500 epochs. The results outperformed that the ERSOCAE-SNC system has achieved effectual classification results. For sample, the ERSOCAE-SNC methodology has recognized samples under OC-F class with $acc{u_y}$, $pre{c_n}$, $rec{a_l}$, ${F_{score}}$, and MCC of 98.62%, 97.94%, 97.93%, 97.93%, and 96.90% correspondingly. Furthermore, the ERSOCAE-SNC algorithm has recognized samples under P-F class with $acc{u_y}$, $pre{c_n}$, $rec{a_l}$, ${F_{score}}$, and MCC of 98.71%, 98.07%, 98.07%, 98.07%, and 97.10% correspondingly. Eventually, the ERSOCAE-SNC method has recognized samples under B-F class with $acc{u_y}$, $pre{c_n}$, $rec{a_l}$, ${F_{score}}$, and MCC of 98.70%, 98.70%, 98.70%, 98.70%, and 97.40% correspondingly.

**Table 4:** Result analysis of ERSOCAE-SNC approach with various class labels under epoch 1,500.

Epoch-500					
Class labels	Accuracy	Precision	Recall	F-score	MCC
Organic carbon-F					
Low	98.20	97.97	96.60	97.28	95.94
Medium	99.33	98.80	99.20	99.00	98.50
High	98.33	97.03	98.00	97.51	96.26
Average	98.62	97.94	97.93	97.93	96.90
Phosphorus-F					
Low	98.80	99.39	98.20	98.79	97.61
Medium	98.80	98.22	99.40	98.81	97.61
Average	98.80	98.81	98.80	98.80	97.61
Potassium-F					
Low	98.13	97.01	97.40	97.21	95.80
Medium	98.93	98.99	97.80	98.39	97.60
High	99.07	98.21	99.00	98.61	97.91
Average	98.71	98.07	98.07	98.07	97.10
Boron-F					
Low	98.70	98.41	99.00	98.70	97.40
Medium	98.70	98.99	98.40	98.70	97.40
Average	98.70	98.70	98.70	98.70	97.40

**Figure 6:**
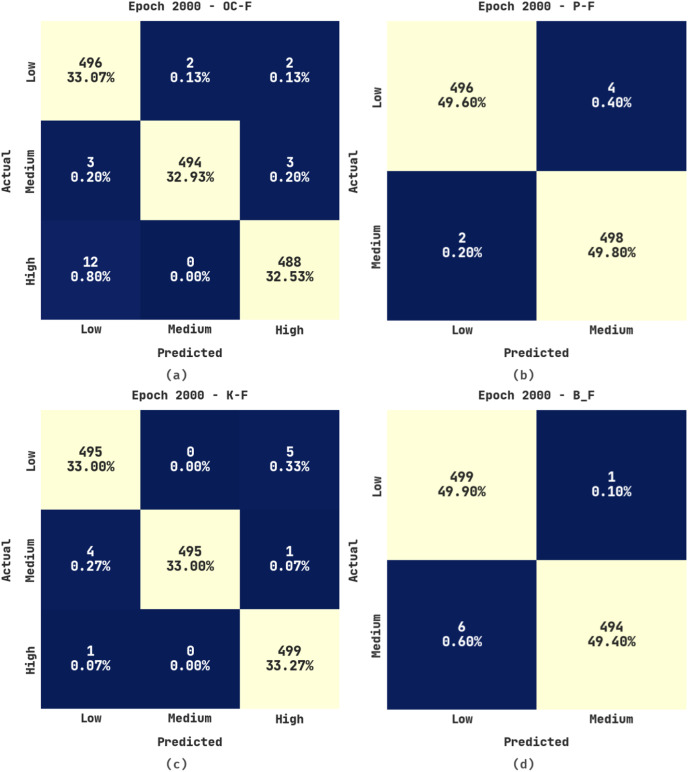
Confusion matrices of ERSOCAE-SNC approach under epoch 2000 (A) OC-F, (B) P-F, (C) K-F, and (D) B_F.

Figure 6 displays the confusion matrices produced by the ERSOCAE-SNC algorithm under 2,000 epochs. With 2,000 epochs, the ERSOCAE-SNC technique has identified the subclasses of OC-F with 33.07% under Low class, 32.93% under medium class, and 32.53% under High class. At the same time, with 2000 epochs, the ERSOCAE-SNC approach has identified the subclasses of K-F with 33% under low class, 33% under medium class, and 33.27% under high class. Concurrently, with 2,000 epochs, the ERSOCAE-SNC methodology has identified the subclasses of B_F with 49.90% under low class and 49.40% under medium class.

Table 5 offers a brief outcome investigation of the ERSOCAE-SNC technique on the soil nutrient classification process with 2,000 epochs. The outcomes exhibited that the ERSOCAE-SNC system has obtained efficient classification results. For sample, the ERSOCAE-SNC system has recognized samples under OC-F class with $acc{u_y}$, $pre{c_n}$, $rec{a_l}$, ${F_{score}}$, and MCC of 99.02%, 98.55%, 98.53%, 98.54%, and 97.81% correspondingly. Along with that, the ERSOCAE-SNC algorithm has recognized samples under P-F class with $acc{u_y}$, $pre{c_n}$, $rec{a_l}$, ${F_{score}}$, and MCC of 99.51%, 99.27%, 99.27%, 99.27%, and 98.90% respectively. Finally, the ERSOCAE-SNC system has recognized samples under B-F class with $acc{u_y}$, $pre{c_n}$, $rec{a_l}$, ${F_{score}}$, and MCC of 99.30%, 99.30%, 99.30%, 99.30%, and 98.60% correspondingly.

**Table 5:** Result analysis of ERSOCAE-SNC approach with various class labels under epoch 2,000.

Epoch-500					
Class labels	Accuracy	Precision	Recall	F-score	MCC
Organic carbon-F					
Low	98.73	97.06	99.20	98.12	97.18
Medium	99.47	99.60	98.80	99.20	98.80
High	98.87	98.99	97.60	98.29	97.45
Average	99.02	98.55	98.53	98.54	97.81
Phosphorus-F					
Low	99.40	99.60	99.20	99.40	98.80
Medium	99.40	99.20	99.60	99.40	98.80
Average	99.40	99.40	99.40	99.40	98.80
Potassium-F					
Low	99.33	99.00	99.00	99.00	98.50
Medium	99.67	100.00	99.00	99.50	99.25
High	99.53	98.81	99.80	99.30	98.96
Average	99.51	99.27	99.27	99.27	98.90
Boron-F					
Low	99.30	98.81	99.80	99.30	98.60
Medium	99.30	99.80	98.80	99.30	98.60
Average	99.30	99.30	99.30	99.30	98.60

**Figure 7:**
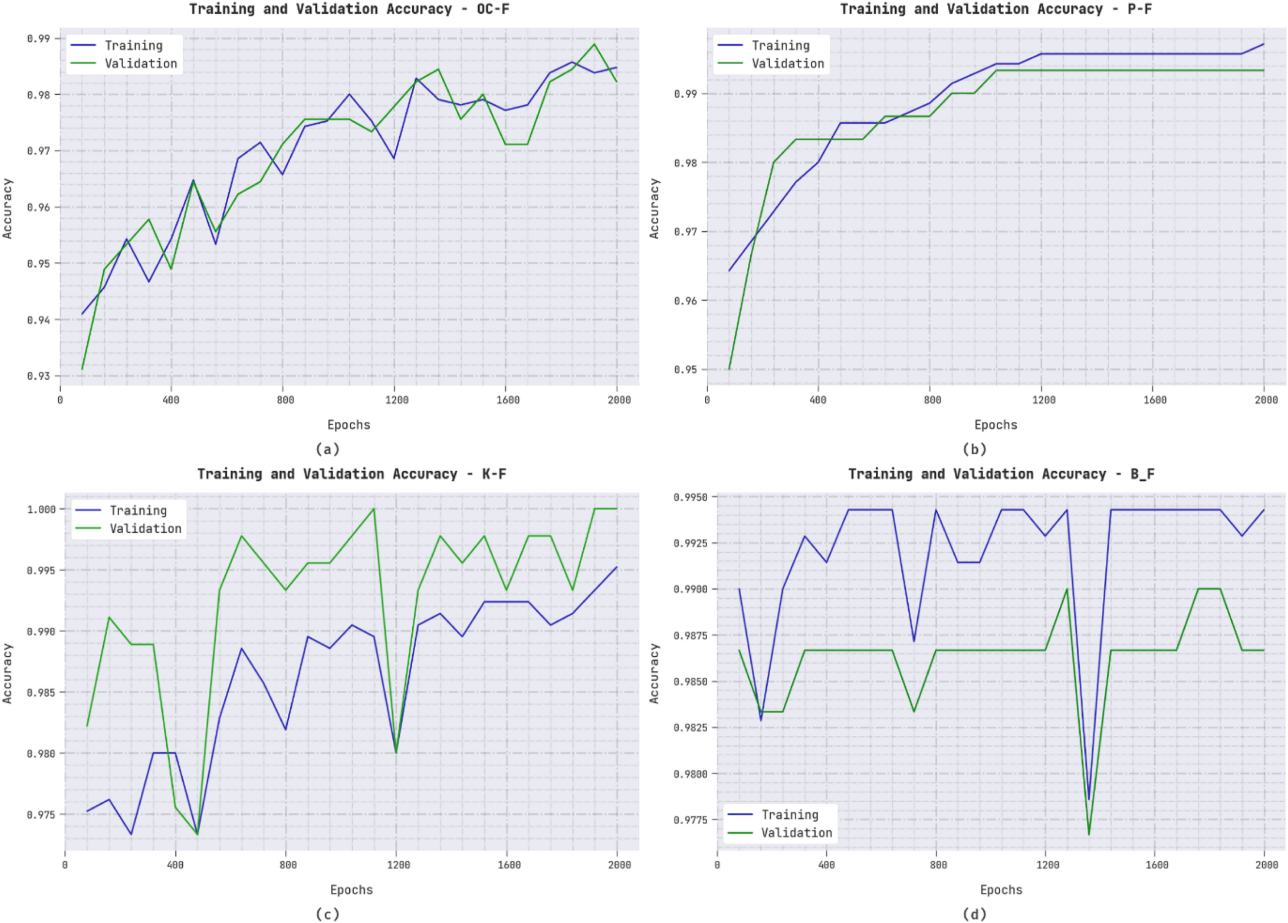
TA and VA analysis of ERSOCAE-SNC approach (A) OC-F, (B) P-F, (C) K-F, and (D) B_F.

The training accuracy (TA) and validation accuracy (VA) acquired by the ERSOCAE-SNC methodology on distinct class labels is depicted in Fig. 7. The experimental outcome inferred that the ERSOCAE-SNC system has attained enhanced values of TA and VA. Particularly the VA executed that superior to TA.

**Figure 8:**
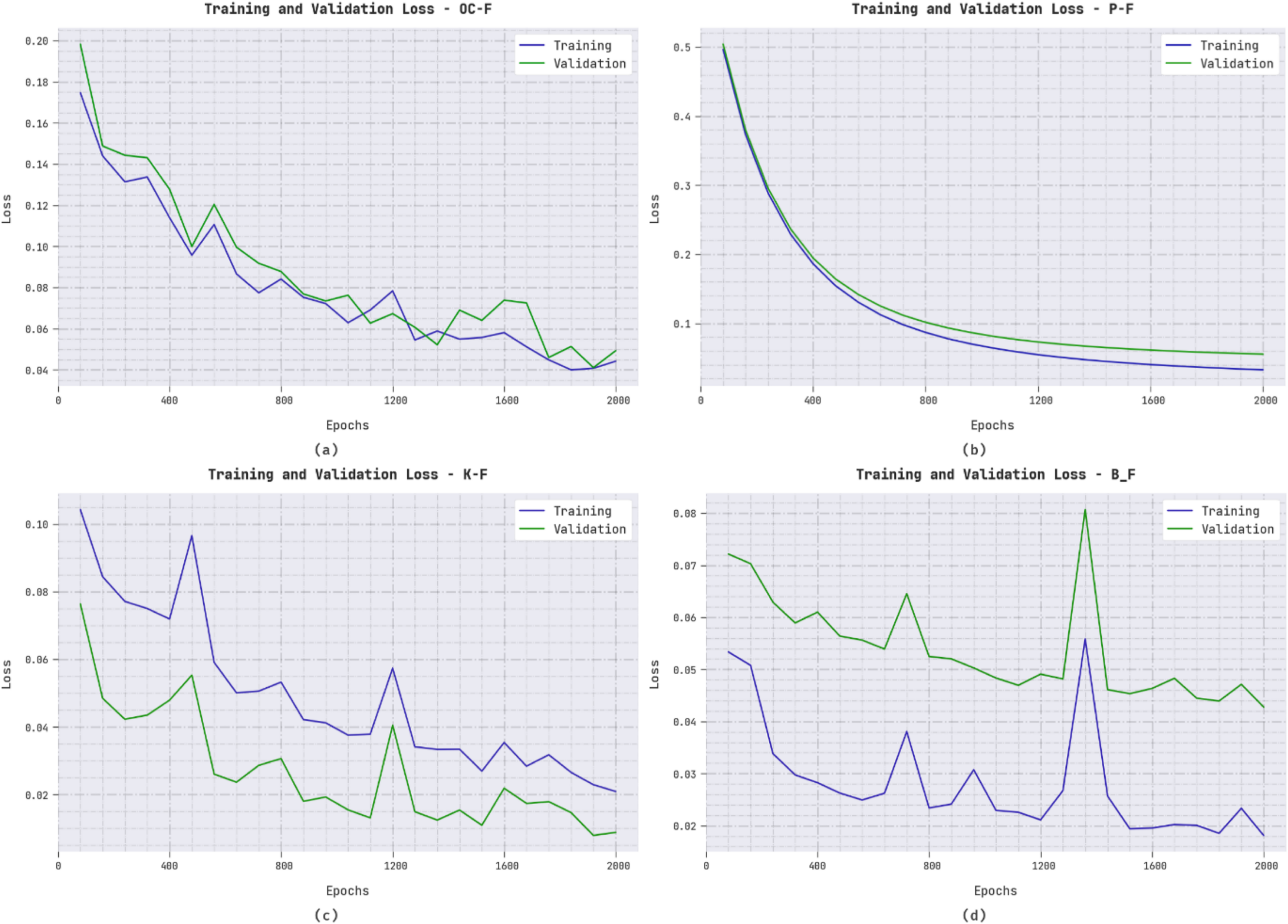
TL and VL analysis of ERSOCAE-SNC approach (A) OC-F, (B) P-F, (C) K-F, and (D) B_F.

The training loss (TL) and validation loss (VL) accomplished by the ERSOCAE-SNC system on distinct class labels are established in Fig. 8. The experimental outcome exposed that the ERSOCAE-SNC algorithm has been able lower values of TL and VL. In specific, the VL is lesser than TL.

**Figure 9:**
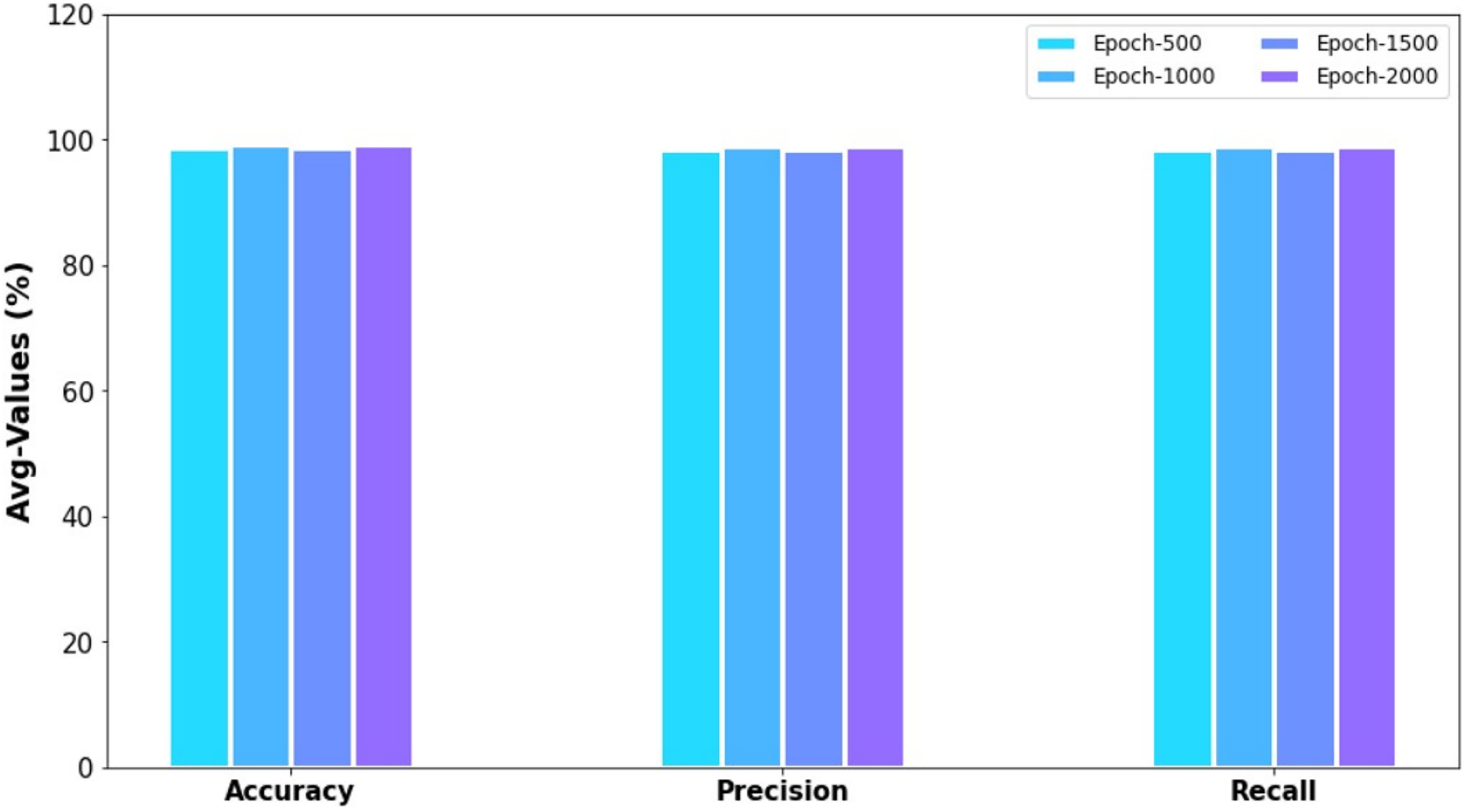
Accu_y_, prec_n_ and raca_l_ analysis of ERSOCAE-SNC approach under distinct epochs.

Table 6 provides an overall result analysis of the ERSOCAE-SNC model under distinct epochs. Figure 9 depicts the average results analysis of the ERSOCAE-SNC model interms of $acc{u_y}$, $pre{c_n}$, and $rec{a_l}$. With 500 epochs, the ERSOCAE-SNC model has offered average $acc{u_y}$, $pre{c_n}$, and $rec{a_l}$ values of 98.77%, 98.41%, and 98.41% respectively. Moreover, with 1,000 epochs, the ERSOCAE-SNC approach has obtainable average $acc{u_y}$, $pre{c_n}$, and $rec{a_l}$ values of 99.16%, 98.93%, and 98.93% correspondingly. Furthermore, with 2,000 epochs, the ERSOCAE-SNC methodology has accessible average $acc{u_y}$, $pre{c_n}$, and $rec{a_l}$ values of 99.31%, 99.13%, and 99.13% correspondingly.

**Table 6:** Overall analysis of ERSOCAE-SNC approach under distinct epochs.

No. of epochs	Accuracy	Precision	Recall	F-score	MCC
Epoch-500	98.77	98.41	98.41	98.41	97.35
Epoch-1000	99.16	98.93	98.93	98.93	98.20
Epoch-1500	98.71	98.38	98.38	98.38	97.25
Epoch-2000	99.31	99.13	99.13	99.13	98.53
Average	98.99	98.71	98.71	98.71	97.83

**Figure 10:**
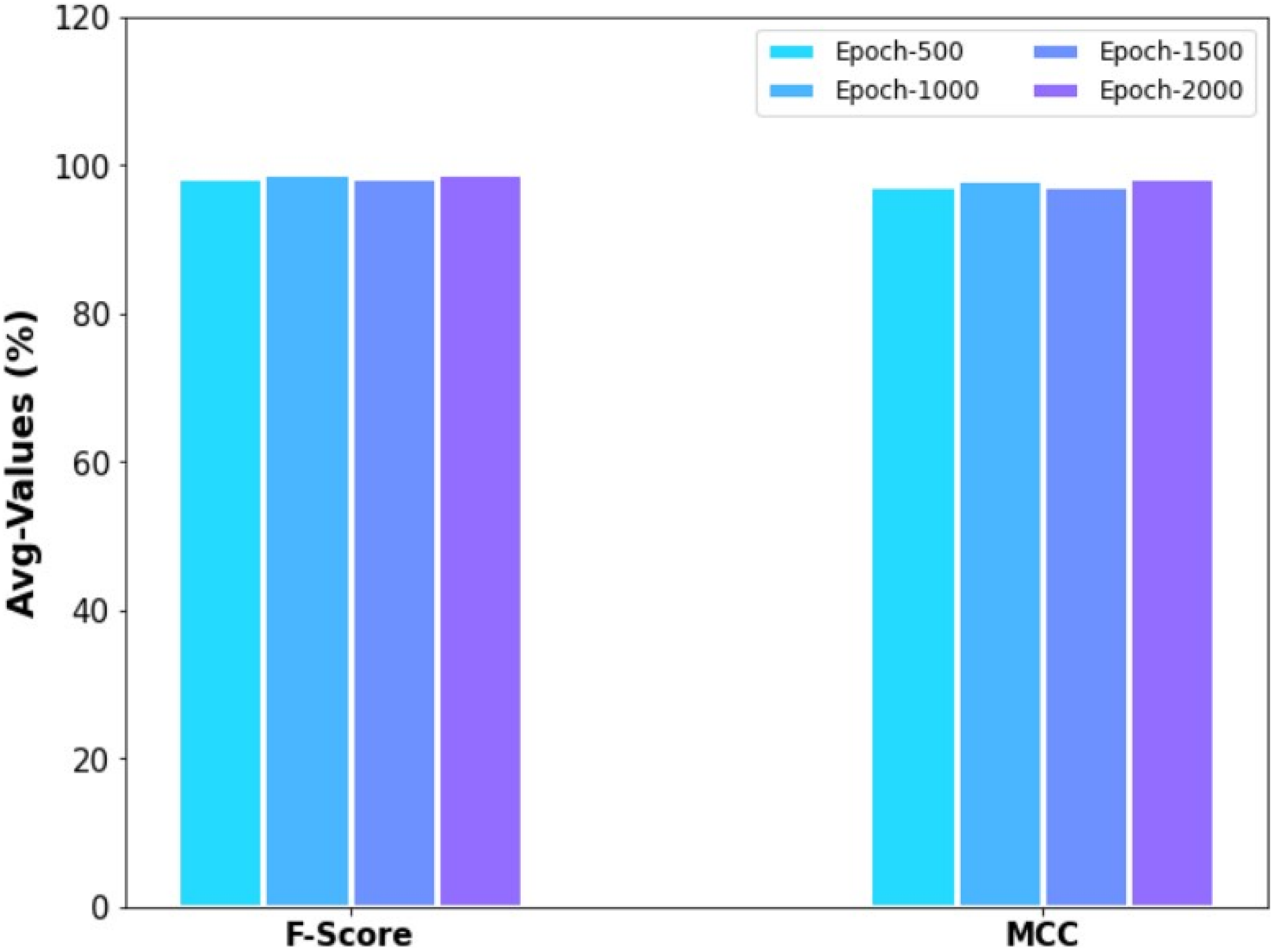
F_score_ and MCC analysis of ERSOCAE-SNC approach under distinct epochs.

Figure 10 demonstrates the average results examination of the ERSOCAE-SNC approach with respect to ${F_{score}}$ and MCC. With 500 epochs, the ERSOCAE-SNC system has obtainable average ${F_{score}}$ and MCC values of 98.41% and 97.35% correspondingly. Also, with 1,000 epochs, the ERSOCAE-SNC system has accessible average ${F_{score}}$ and MCC values of 98.93% and 98.20% correspondingly. At last, with 2,000 epochs, the ERSOCAE-SNC approach has accessible average ${F_{score}}$ and MCC values of 99.13% and 98.53% correspondingly.

## Discussion

**Figure 11:**
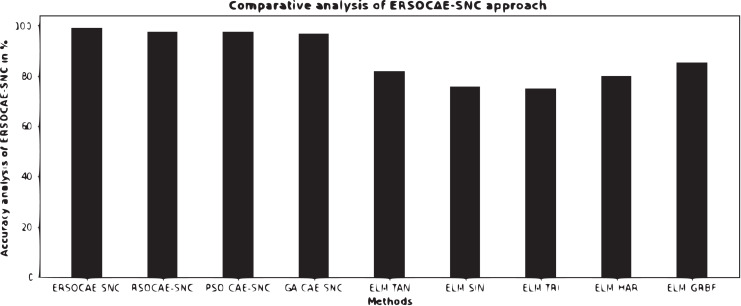
Comparative analysis of ERSOCAE-SNC approach with existing methodologies.

To assure the enhanced performance of the ERSOCAE-SNC model, a detailed comparison study is made in Table 7 and Fig. 11. The experimental results ensured that the ELM-SIN and ELM-TRI models have demonstrated lower $acc{u_y}$ values of 75.49% and 74.83% respectively.

**Table 7:** Comparative analysis of ERSOCAE-SNC approach with existing methodologies.

Methods	Accuracy (%)
ERSOCAE-SNC	98.99
RSOCAE-SNC	97.46
PSO-CAE-SNC	97.31
GA-CAE-SNC	96.82
ELM-TAN	81.86
ELM-SIN	75.49
ELM-TRI	74.83
ELM-HAR	79.90
ELM-GRBF	85.08

At the same time, the ELM-TAN and ELM-HAR models have shown slightly improved $acc{u_y}$ values of 81.86% and 79.90% respectively. Meanwhile, the ELM-GRBF model has exhibited reasonable $acc{u_y}$ of 85.08%. At the same time, the RSOCAE-SNC, PSO-CAE, SNC, and GA-CAE-SNC models have reported closer $acc{u_y}$ of 97.46%, 97.31%, and 96.82% respectively. However, the ERSOCAE-SNC model has resulted in maximum $acc{u_y}$ of 98.99%.

**Figure 12:**
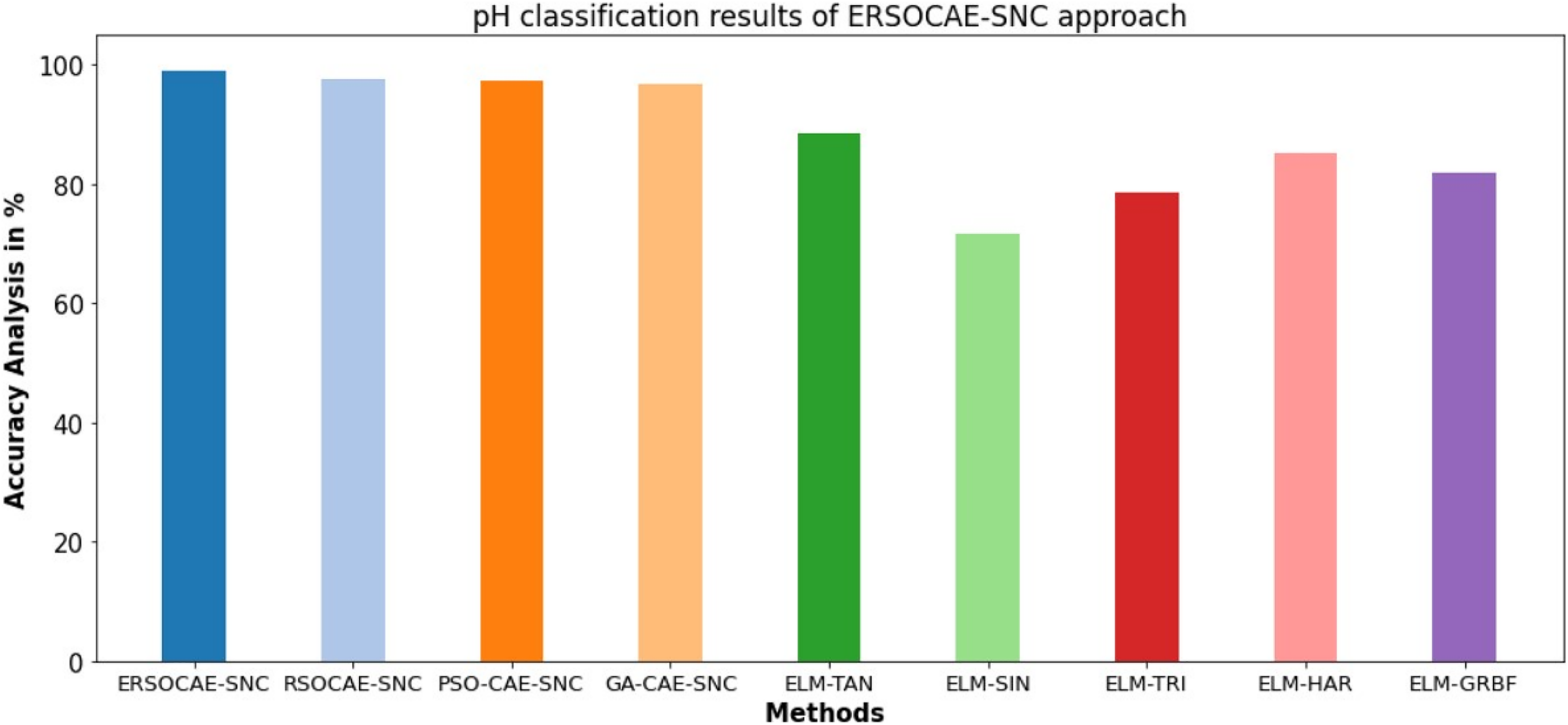
pH classification results of ERSOCAE-SNC approach with existing methodologies.

Finally, a detailed pH classification results of the ERSOCAE-SNC model with recent models take place in Table 8 and Fig. 12. The experimental outcomes stated that the ELM-SIN and ELM-TRI models have reported lower $acc{u_y}$ of 71.74% and 78.52% respectively. Followed by, the ELM-TAN, ELM-HAR, and ELM-GRBF models have accomplished closer $acc{u_y}$ of 88.59%, 85.23%, and 81.87% respectively.

**Table 8:** pH classification results of ERSOCAE-SNC approach with existing methodologies.

Methods	Accuracy (%)
ERSOCAE-SNC	99.12
RSOCAE-SNC	97.68
PSO-CAE-SNC	97.47
GA-CAE-SNC	96.87
ELM-TAN	88.59
ELM-SIN	71.74
ELM-TRI	78.52
ELM-HAR	85.23
ELM-GRBF	81.87

Though the RSOCAE-SNC, PSO-CAE-SNC, and GA-CAE-SNC models have reported reasonable $acc{u_y}$ values of 97.68%, 97.47%, and 96.87%, the ERSOCAE-SNC model has shown maximum $acc{u_y}$ of 99.12%. From the detailed results and discussion, it is apparent that the ERSOCAE-SNC model has accomplished maximum soil nutrient classification performance over other models.

## Conclusion

The loss of fertility in soil leads to the lack of productivity. The availability of soil nutrients to crop plants and nutrients availability depends on soil pH. Therefore, the classification of soil nutrients and pH indices help to save the time of soil experts to analyses soil health and environmental quality. In this study, a new ERSOCAE-SNC approach was established for soil nutrient classification. The presented ERSOCAE-SNC method aimed to categorize the nutrient levels of P, K, OC, and B and soil pH. To accomplish this, the ERSOCAE-SNC model applied a three-stage process namely sample collection, CAE classification, and ERSO based hyperparameter optimization. As the trial-and-error method for hyperparameter tuning of CAE model is a tedious and erroneous process, the ERSO algorithm has been utilized which in turn enhances the soil nutrient classification performance. The proposed work has a limitation in that the level of compression is dependent on the CAE’s architecture and, more specifically, the encoder output. It should be noted that the higher the level compression, the smaller the encoder’s output. Besides, the ERSO algorithm is derived by incorporating the chaotic concepts into the RSO algorithm. To assuring the improved performance of the ERSOCAE-SNC model, an extensive ranging experimental study was executed and outcomes were inspected under distinct aspects. The experimental findings demonstrated the ERSOCAE-SNC technique’s superior performance to more recent techniques, with an accuracy of 98.99% for soil nutrients and 99.12% for soil pH respectively. By properly classifying soil nutrients and pH, the proposed model can be used as a useful tool to increase agricultural productivity. The comparative study highlighted the superior performance of the ERSOCAE-SNC model over other approaches. This model may help the Tamil Nadu government to make effective decisions in improving the quality of the soil and crop production. In the future, applications of the ERSOCAE-SNC technique could automate agricultural processes in a real-time setting. In addition, by combining a hybrid metaheuristic optimization process with a feature selection process, the performance of the proposed model can be enhanced. Additionally, future studies of the proposed model’s performance on huge datasets are possible.

## Supplemental Information

10.7717/peerj.15147/supp-1 Supplemental Information 1 Soil nutrient classification dataset for ERSOCAE-SNC model.

## Additional Information and Declarations

### Data Availability

The following information was supplied regarding data availability: The data is available at Kaggle: https://www.kaggle.com/code/meghaljambhale/soil-fertility-eda-and-model-analysis/data.
